# The extent of extra-axonal tissue damage determines the levels of CSPG upregulation and the success of experimental axon regeneration in the CNS

**DOI:** 10.1038/s41598-018-28209-z

**Published:** 2018-06-29

**Authors:** Juhwan Kim, Muhammad S. Sajid, Ephraim F. Trakhtenberg

**Affiliations:** 1 Department of Neuroscience, University of Connecticut School of Medicine, 263 Farmington Ave., Farmington, CT 06030 USA; 2 University of Hartford, 200 Bloomfield Ave., West Hartford, CT 06117 USA

## Abstract

The failure of mature central nervous system (CNS) projection neurons to regenerate axons over long distances drastically limits the recovery of functions lost after various CNS injuries and diseases. Although a number of manipulations that stimulate some degree of axon regeneration that overcomes the inhibitory environment after CNS injury have been discovered, the extent of regeneration remains very limited, emphasizing the need for improved therapies. Regenerating axons need nerve tissue environment capable of supporting their growth, and severe extra-axonal tissue damage and remodeling after injury may disrupt such environment. Here, we used traumatic injury to the mouse optic nerve as a model system to investigate how the extent of extra-axonal tissue damage affects experimental axon regeneration. Axon regeneration was stimulated by the shRNA-mediated knockdown (KD) of Pten gene expression in the retinal ganglion cells, and the extent of extra-axonal tissue damage was varied by changing the duration of optic nerve crush. Although no axons were spared using either 1 or 5 seconds crush, we found that Pten KD-stimulated axon regeneration was significantly reduced in 5 seconds compared with 1 second crush. The more severe extra-axonal tissue damage did not cause tissue atrophy, but led to significantly higher upregulation of axon growth-inhibiting chondroitin sulfate proteoglycan (CSPG) in the glial scar and also enlarged glial scar size, compared with less severely damaged tissue. Thus, the success of axon-regenerating approaches that target neuronal intrinsic mechanisms of axon growth is dependent on the preservation of appropriate extra-axonal tissue environment, which may need to be co-concurrently repaired by tissue remodeling methods.

## Introduction

The failure of spontaneous long-distance axon regeneration in mammalian central nervous system (CNS) projection neurons after axonal injury has devastating consequences for those who sustained spinal cord injury^1^, stroke^2,3^, brain trauma^4,5^, and optic neuropathy^6–9^. Because spontaneous axon regeneration failure in the CNS affects mammals, but not necessarily lower vertebrates, rodent models of optic nerve, spinal cord, and brain injuries have been developed to tackle this problem. For example, like other non-retinal CNS projection neurons, rodent retinal ganglion cells (RGCs) do not spontaneously regenerate axons disrupted by an optic nerve crush (ONC) injury^10,11^. Although modest sprouting near the injury site may occur, the axons do not regenerate over long distances without treatment. Importantly, molecules found to regulate regeneration of RGC axons, such as Pten and Klf7^12,13^, were also found to affect spinal cord regeneration^14,15^. These findings support the hypothesis that the process of axonal growth and regeneration *per se* may involve similar mechanisms across CNS projection neurons, while their mechanisms of pathway finding vary.

A number of intracellular and extracellular factors have been discovered to affect axon regeneration, as reviewed elsewhere^8,16–21^, but full-length regeneration that can lead to recovery of even simple visual functions^22,23^ involves manipulation of tumorigenic factors, which may be too risky for clinical use in humans^24^. Nevertheless, these studies have shown that, in principle, stimulating neuronal intrinsic mechanisms of axon regeneration alone could be sufficient for therapeutic recovery of function, bypassing the need to co-regulate guidance cues, attenuate extracellular inhibitors, or promote synaptogenesis; although such complementary treatments may be helpful in further improving outcomes. Even with an ability to overcome extracellular inhibitors and other challenges to regeneration, the success of such approaches may depend on preservation of the extra-axonal tissue environment^25^, which is needed to facilitate the process of axon regeneration by providing substrate for growth, guidance cues, support cells, and vascularization. Therefore, although therapeutic tissue remodeling is needed to help those who sustained more severe injuries, investigations into neuronal capacity for regenerating axons should not be confounded by extensive damage to extra-axonal tissue, because it limits our ability to appropriately evaluate the therapeutic potential of factors that can promote axon regeneration and help individuals without severe extra-axonal tissue damage. Ultimately, we envision co-treatment with tissue remodeling and axon regeneration therapies to help those who suffered more severe injuries as well.

Here, we investigated how the extent of extra-axonal tissue damage affects experimental axon regeneration. We found that more severe damage to the extra-axonal tissue reduces axon regeneration stimulated by knockdown (KD) of Pten in RGCs. We also found that although more severe damage did not increase tissue atrophy, the level of upregulation of axon regeneration-inhibiting CSPG presented by reactive astrocytes^26–28^ and an increase in the glial scar size^20^, are correlated with the extent of extra-axonal tissue damage. We also demonstrate how inefficient ONC could lead to axonal sparing and describe the ways to control for this issue.

## Results

**Figure 1:**
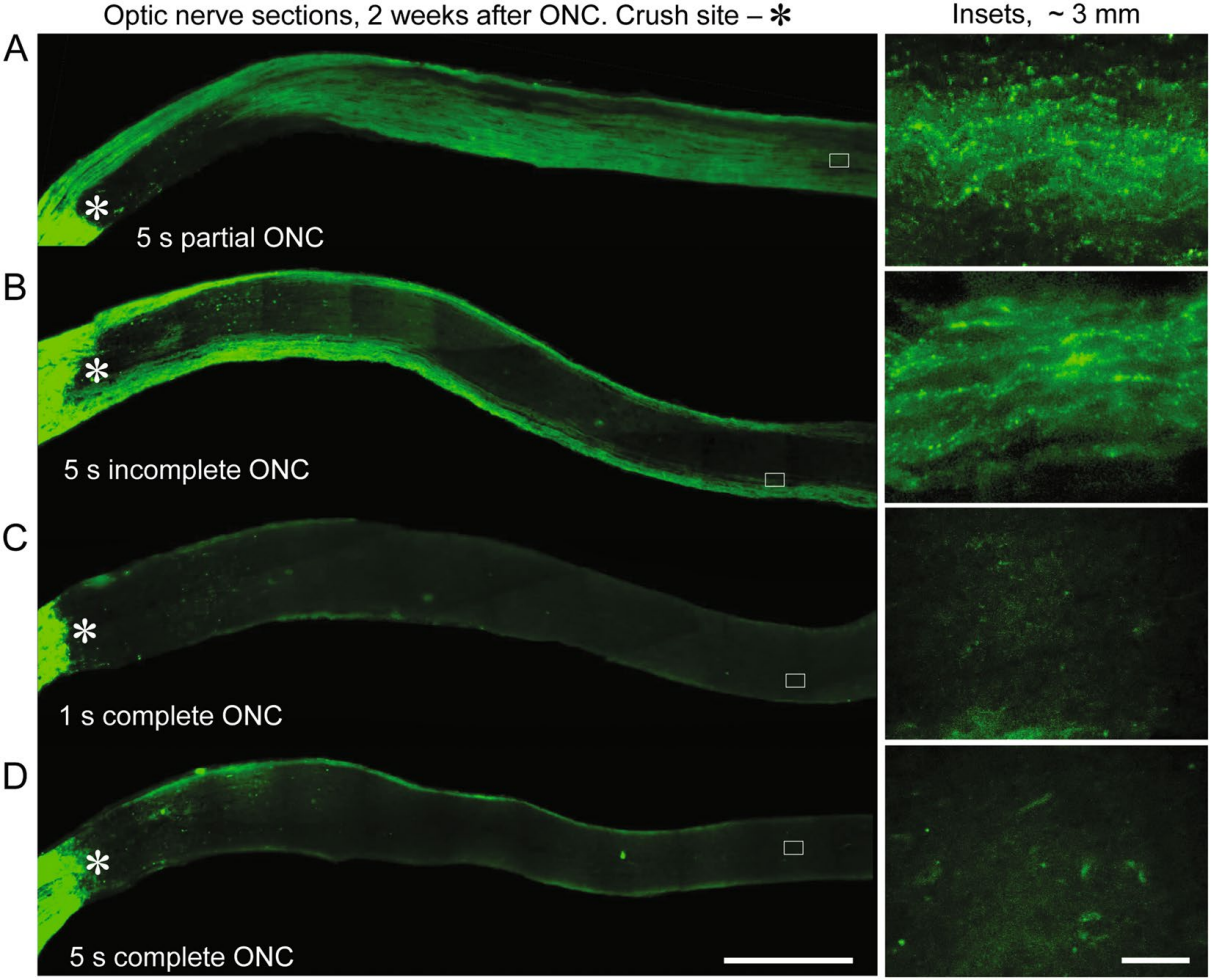
Pitfalls in experimental ONC injury that lead to axonal sparing, and absence of spontaneous axon regeneration or axoplasmic recovery after complete ONC. Representative images of the optic nerve longitudinal sections with CTB-labeled axons through the optic nerve, 2 weeks after ONC that was performed with various adjustments, as marked. (**A**) Partial ONC, in which only half of the optic nerve width was grasped with the tips of forceps used to perform the injury, and the duration of the pinch was 5 seconds. (**B**) Incomplete ONC, in which full-width of the optic nerve is grasped but the forceps’ tips were not closed completely during 5 seconds pinch. (**C**,**D**) Complete ONC, in which full-width of the optic nerve is grasped and the forceps’ tips closed completely during 1 (C) or 5 (D) seconds pinch. The edges of the tissue were cropped out due to artefactual autofluorescence that is common at tissue edges. Insets: Representative images of the optic nerve regions distal from the injury site are magnified for better visualization of the axons or their absence. Scale bars, 500 µm (main panels), 10 µm (insets).

**Figure 2:**
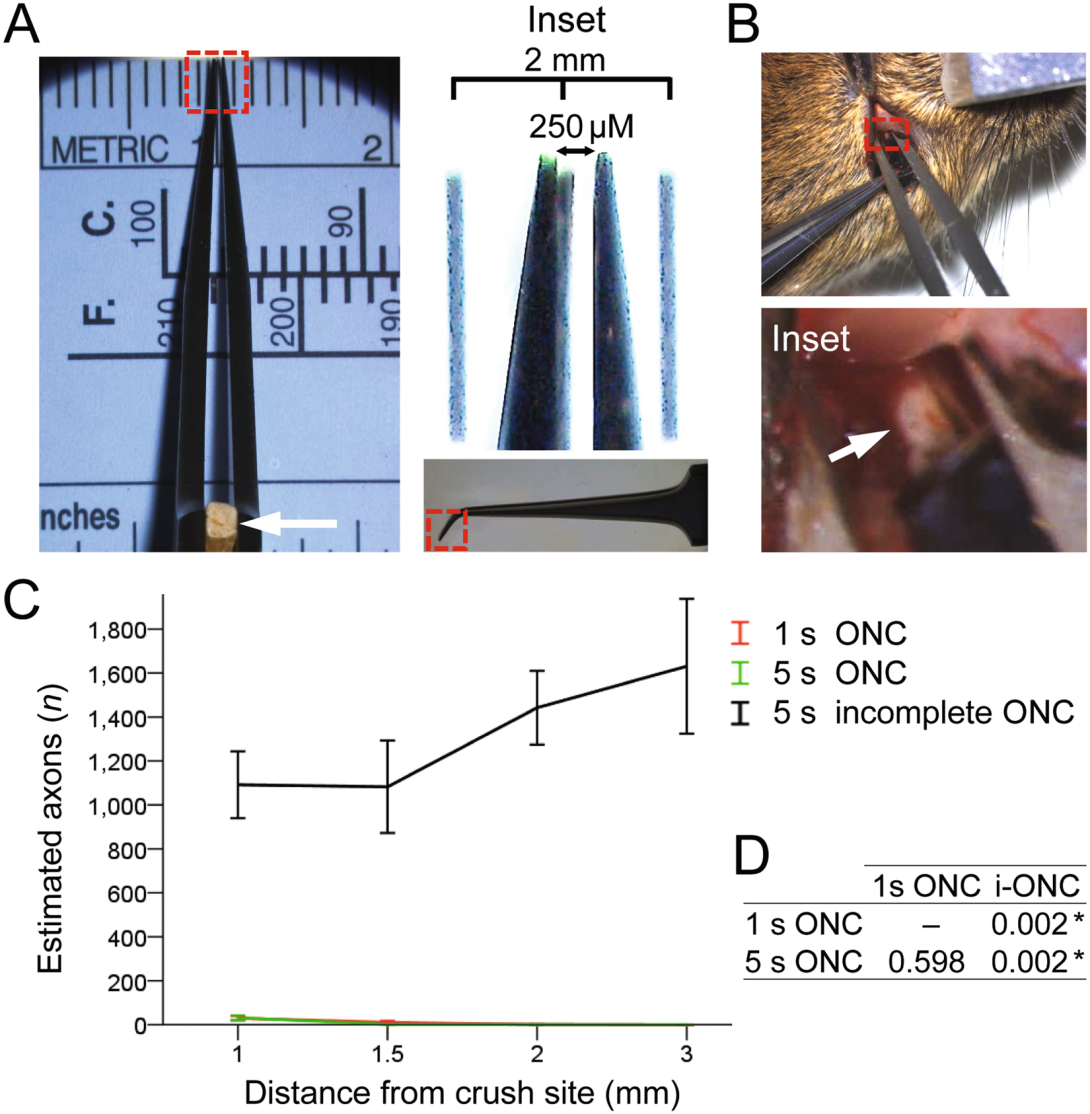
Quantitation of axons after incomplete and complete experimental ONC injuries. (**A**) Toothpick fragment (arrow) is glued to one side of the forceps (*main panel*), at a distance from the tips such that when closed, a 250 µm gap remains between the tips (*inset*). (**B**) To visually confirm a complete crush, a conjunctival incision is made (in anesthetized mice) over the dorsal (*main panel*), rather than lateral aspect of one eye, and the orbital muscles are slightly separated to expose the optic nerve (arrow in *inset*), which is then pinched (it is more difficult to visually confirm a complete grasp of the optic nerve when making an incision over the lateral aspect of an eye). (**C**,**D**) Quantitation of axons along the optic nerve at increasing distances from the injury site, 2 weeks after ONC that was performed with various adjustments, as marked (mean ± S.E.M; *n* = 4 cases for each group) (C). Analysis by ANOVA with repeated measures, sphericity assumed, overall *F* = 98.2, *p* < 0.0001; *p*-values of pairwise comparisons by posthoc LSD and significant differences indicated by an asterisk (D).

In order to evaluate axon regeneration after ONC reliably, experimental injury needs to disrupt all the axons within the optic nerve. Inefficient ONC can lead to axonal sparing, which can confound the results^29^. However, excessively harsh injury may lead to unnecessary extra-axonal tissue damage, which we show in this study impedes regeneration and confounds the results (see below). Therefore, it is important to determine a balanced approach, which disrupts all axons but does not cause excessive extra-axonal tissue damage. Inefficient ONC can result from the tips of the forceps used to crush the optic nerve not grasping the full-width of the nerve tissue (Fig. 1A), or from incomplete closing of the tips during the nerve “pinch” even if its full-width is grasped (Figs 1B and 2A,C,D). We find that when an appropriate grasp is visually confirmed (Fig. 2B) and the pinch is complete (i.e., force sufficient to close the tips without the nerve is also applied when the nerve is pinched), all the axons are disrupted irrespective of whether pinch duration is 1 or 5 seconds (Figs 1C,D and 2C,D). While spontaneous axoplasmic recovery has been shown in a glaucoma model when increased intraocular pressure (IOP) was normalized^30^, in the ONC model even 2 weeks after injury, only rare modest sprouting (up to 1 mm distance) from the injury site was detected and it was not significantly different (*p* = 0.6) between 1 or 5 seconds ONC (Figs 1C,D and 2C,D). The absence of any intact axons distal from injury sites along the optic nerve even 2 weeks after ONC demonstrates that all the axons were disrupted, and therefore, axoplasmic recovery does not occur in this model when performed as above.

**Figure 3:**
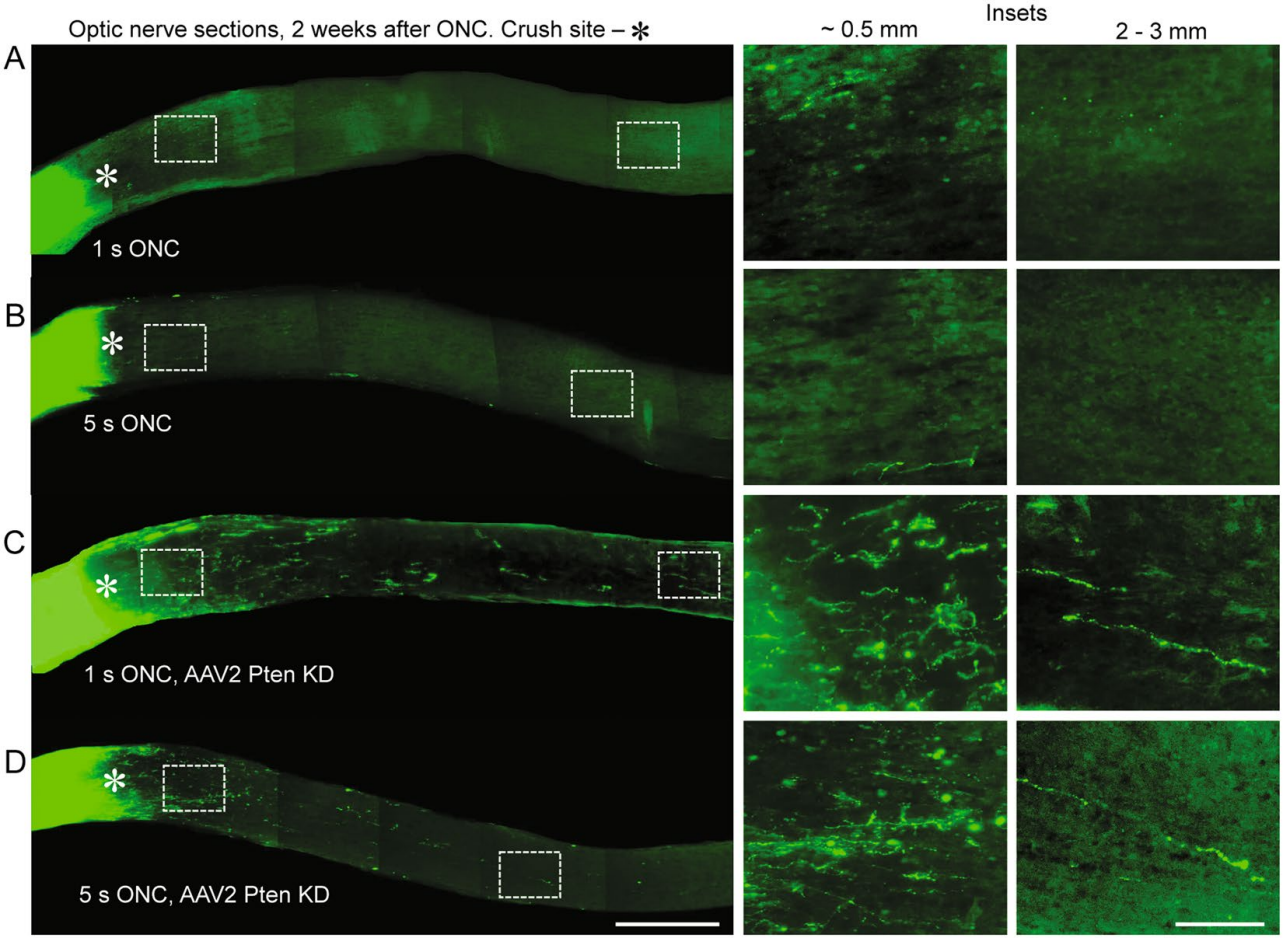
Axon regeneration stimulated by Pten KD after 1 s or 5 s ONC. (**A,B**) Representative images of the optic nerve longitudinal sections with CTB-labeled axons through the optic nerve, 2 weeks after 1 s (*A*) or 5 s (*B*) ONC. (**C**,**D**) Representative image of the optic nerve longitudinal sections with CTB-labeled axons regenerating through the optic nerve, 2 weeks after 1 s (C) or 5 s (D) ONC and pre-treatment with anti-Pten shRNA AAV2. The edges of the tissue were cropped out due to artefactual autofluorescence that is common at tissue edges. Insets: Representative images of the optic nerve regions proximal and distal to the injury site are magnified for better visualization of the axons or their absence. Scale bars, 500 µm (main panels), 100 µm (insets).

**Figure 4:**
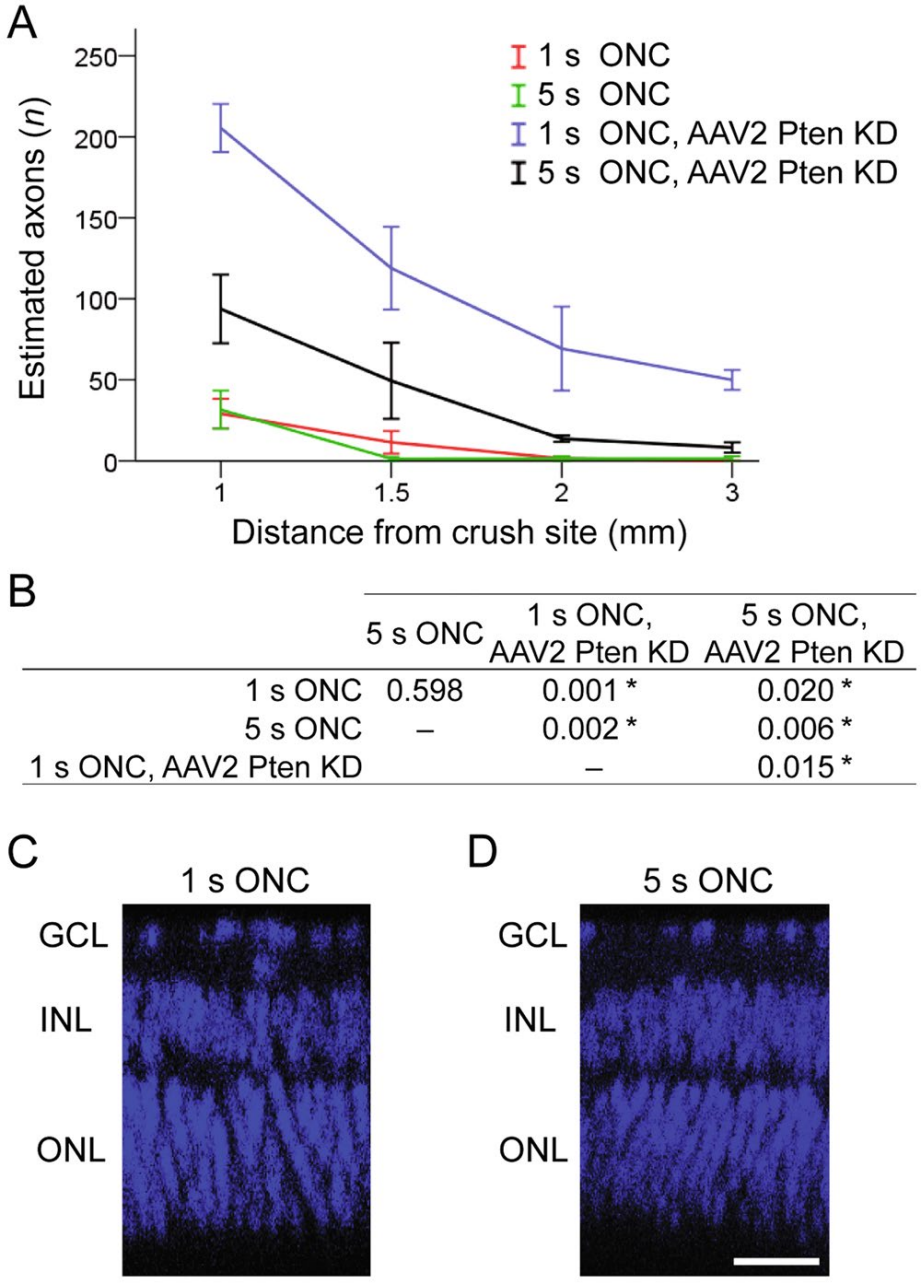
Quantitation of axon regeneration and retinal tissue thickness 2 weeks after 1 s and 5 s ONC. (**A**) Quantitation of axons along the optic nerve at increasing distances from the injury site, 2 weeks after 1 s or 5 s ONC and pre-treatment with anti-Pten shRNA AAV2, as marked (mean ± S.E.M; *n* = 4 cases for each group. (**B**) Analysis by ANOVA with repeated measures, sphericity assumed, overall *F* = 63.2, *p* < 0.0001; *p*-values of pairwise comparisons by posthoc LSD and significant differences indicated by an asterisk. (**C**,**D**) Representative images of the retinal tissue thickness from untreated animals at 2 weeks after 1 s (C) or 5 s (D) ONC, visualized by DAPI labeling of retinal layers, as marked. GCL, ganglion cell layer; INL, inner nuclear layer; and ONL, outer nuclear layer. Scale bar, 25 µm.

Next, we investigated whether severity of damage to extra-axonal tissue affects experimental axon regeneration. KD of Pten in RGCs using intravitreally injected anti-Pten shRNA AAV2 vector is a well-established method for stimulating axon regeneration after ONC^13,31–33^. Therefore, we used this method in adult mice, and 2 weeks later performed 1 or 5 seconds ONC, as above, in randomly selected treated and untreated animals. Because even 1 second ONC is sufficient to disrupt all the axons (Figs 1 and 2**)**, we hypothesized that increasing duration of the injury to 5 seconds will affect primarily extra-axonal tissue (since all the axons are disrupted by 1 second injury), and that ensuing excessive extra-axonal tissue damage will hinder axon regeneration stimulated by Pten KD in RGCs. To visualize regenerating axons, anterograde axonal tracer cholera toxin subunit B (CTB) was intravitreally injected 1 day before animals were euthanized 2 weeks after ONC. The number of regenerating axons was quantified in longitudinal sections of the optic nerve, following an established method we used previously^24^ (also described in the Methods). We found that while Pten KD stimulated axon regeneration after 1 and 5 seconds ONC, in the group that had 1 second ONC axon regeneration was significantly (*p* < 0.02) more robust compared with the group that had 5 seconds ONC (Figs 3 and 4**)**. Both, the number of regenerating axons and the distance they grew were greater in the 1 second compared with 5 seconds ONC group (both treated with anti-Pten shRNA). No spared axons were detected in either group. These data support our hypothesis that excessive damage to extra-axonal tissue reduces axon regeneration stimulated by targeting neuronal intrinsic mechanisms of axon growth.

**Figure 5:**
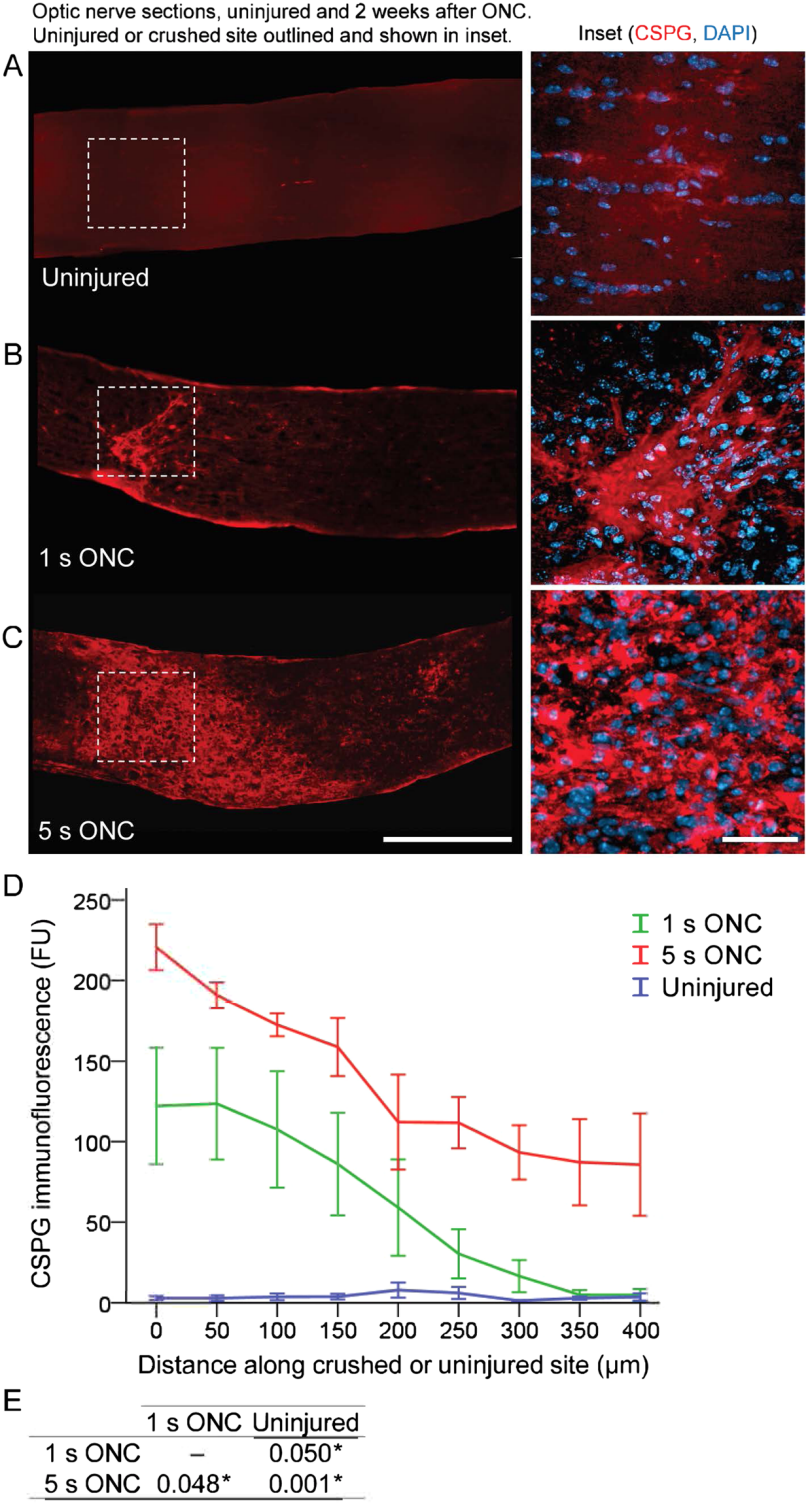
CSPG upregulation and the glial scar after 1 s and 5 s ONC. (**A**–**C**) Representative images of the optic nerve longitudinal sections immunostained for CSPG in uninjured nerve (A), or 2 weeks after 1 s (B) or 5 s (C) ONC. Insets: Representative images of the uninjured or crushed site optic nerve regions are magnified for better visualization of CSPG signal pattern, along with DAPI to label nuclei. Scale bars, 200 µm (main panels), 50 µm (insets). (**D**,**E**) Quantitation of CSPG immunofluorescence signal intensity, represented in fluorescent units (FUs), at increasing distances along the optic nerve injury site at 2 weeks after ONC, or in an equivalent region of the uninjured optic nerve, as marked (mean ± S.E.M; *n* = 4 cases for each group) (D). Analysis by ANOVA with repeated measures, sphericity assumed, overall *F* = 26.5, *p* < 0.001; *p*-values of pairwise comparisons by posthoc LSD and significant differences indicated by an asterisk (E).

We then investigated the mechanisms that may underlie the inhibitory effect of excessive extra-axonal tissue damage on experimental axon regeneration. Excessive tissue damage may lead to atrophy, which in turn could distort and limit the environment available for axons to regenerate through. First, we compared thickness of the retina, where RGC soma reside, to test for potential differences in retinal tissue degeneration that may follow optic nerve injury. We did not find significant differences in the retinal tissue thickness between 1 and 5 seconds ONC groups (Fig. 4C,D; *p* = 0.87 by *t*-test, 2-tailed; *n* = 4 per group). Next, we measured and compared the width of optic nerve tissue at various distances along the optic nerves. There were no significant differences in the widths of the optic nerves between 1 and 5 seconds ONC groups (*p* = 0.85, *n* = 4 per group; compared at increasing distances from the injury site along the longitudinal optic nerve sections, by Repeated Measures ANOVA with posthoc LSD). Reactive astrocytes in the glial scar that forms after nerve injury upregulate CSPG molecules, which inhibit axon regeneration^26–28^. Thus, we hypothesized that excessive tissue damage may lead to formation of a larger glial scar and higher upregulation of CSPG molecules, which may present a more inhibitory environment that fewer regenerating axons could overcome. To test this hypothesis, we immunostained longitudinal optic nerve sections for CSPG and quantified immunofluorescent signal intensities at increasing distances along the injury site or in the same region of an uninjured optic nerve (Fig. 5A–C). We found that CSPG was significantly (*p* < 0.05) upregulated after 5 seconds compared to 1 second ONC (Fig. 5D,E), and the CSPG-positive area was also larger after 5 seconds compared with 1 second ONC. Taken together, these data suggest that although more severe ONC injury did not cause apparent extra-axonal tissue atrophy, it did lead to higher upregulation of axon growth-inhibiting CSPG and a larger glial scar, compared with less severe extra-axonal injury (which is also sufficient to disrupt all the axons in the optic nerve). Thus, reduced axon regeneration we found after 5 seconds compared with 1 second ONC could be due to more severe extra-axonal injury causing a more inhibitory environment for axon growth, as part of the tissue remodeling process that forms a glial scar.

## Discussion

No clinical treatments are available to date to help those who suffer from loss of function due to axonal injuries associated with spinal cord injury^1^, stroke^2,3^, brain trauma^4,5^, and optic neuropathy^6–9^. The ONC rodent model of traumatic optic neuropathy (TON) is a well-established system for tackling the fundamental problem of long-distance axon regeneration failure in the CNS and for determining therapeutic potential of novel treatments^10,11^. For example, molecules such as Pten and Klf7 that were discovered to control axon growth in this model^12,13^, were later found to also regulate spinal cord axon regenereation^14,15^. Thus, although different types of CNS projection neurons vary in their mechanisms of pathway finding, the process of their axonal growth and regeneration *per se* may involve similar mechanisms. Importantly, prior studies have demonstrated that, in principle, stimulating regeneration of axons alone could be sufficient for overcoming the extracellular inhibitors and therapeutic recovery of function^22,23^, bypassing the need to co-regulate guidance cues, attenuating extracellular inhibitors, or promoting synaptogenesis; although such complementary treatments may be helpful in further improving outcomes. While these studies were groundbreaking in demonstrating that in principle therapeutic axon regeneration is possible, manipulation of tumorigenic factors they involved may be too risky for clinical use in humans^24^, and therefore, there is a need to develop safer therapeutic approaches, such as those we have tested in a recent study^24^.

In the present study, we demonstrated that the success of experimental axon regeneration that targets neuronal intrinsic mechanisms depends on the preservation of an extra-axonal tissue environment, suggesting that it is needed for supporting the process of axon regeneration. We found higher CSPG upregulation and a larger glial scar, along with an associated reduction of axon regeneration, after 5 seconds compared with 1 second ONC. Although the Pten KD we used is sufficient for enabling axons to overcome the inhibitory extracellular environment after injury, the success of this approach is very limited as only a small subset of RGC axons regenerated and even those stalled growth far before reaching their targets. Our findings suggest that, the extent of experimental regeneration may be determined by the concentration and distribution of inhibitory molecules that are upregulated in response to, and proportional to the severity of, nerve injury. These findings raise the hypothesis that, axons stimulated to regenerate by approaches such as KD of Pten, may be able to overcome only limited levels of the inhibitory extracellular molecules. Consequently, even when the axons pass through/around the glial scar, the continuous interaction of the proximal axonal segment with the inhibitory molecules associated with the glial scar may reach a threshold that ultimately stalls growth.

In addition to higher upregulation of extracellular inhibitory molecules such as CSPG in formation of a larger glial scar that limits axon regeneration^26–28^, extensive damage to extra-axonal tissue may also disrupt the guidance cues along the pathway through which axons need to regenerate. Other factors associated with glial cells, ECMs, and inflammation may also respond differently to a more severe extra-axonal injury, including molecules that are needed to support axon regeneration^18,25,34,35^. Along with traumatic injury, harsher damage to the ophthalmic and central retinal blood vessels could cause a more severe ischemic oxygen deprivation in the injured region of the optic nerve, which may be needed to support the regenerating axons^9,36–38^. Because ischemic and traumatic optic nerve injuries lead to ATP release, which can in turn activate astrocytes^39–42^, it is possible that an increase in CSPG upregulation by reactive astrocytes in our studies was partially due to increased purinergic signaling. While we did not find significant differences in retinal thickness after 1 or 5 seconds ONC at 2 weeks after injury, such differences may manifest later, as retinal degeneration progresses over several weeks after traumatic and ischemic optic nerve injuries.

Our findings suggest that, studies aimed at investigating neuronal capacity for regenerating axons should not be confounded by extensive damage to extra-axonal tissue because it limits the ability to appropriately evaluate therapeutic potential of factors, which can promote axon regeneration *per se* and help individuals whose axons are disrupted without accompanying severe extra-axonal tissue damage. Considering complex milieu of the extra-axonal post-injury environment^25,34^, our findings also highlight the importance of investigating how extent of an injury differentially affects various other extra-axonal factors that may play a role in regeneration. Ultimately, we envision co-treatment with tissue remodeling and axon regeneration therapies helping those who suffered more severe injuries as well. To avoid confounding excessive damage to extra-axonal tissue, ONC should not be too harsh (although harsher injury may be appropriate for studying RGC survival^43,44^). This may potentially lead to axonal sparing, if experimental injury is not performed appropriately^29^. In this study, we demonstrate the ways in which inefficient ONC could lead to axonal sparing, as well as how to control for this issue without needing excessively harsh injury. Such a balanced approach, which disrupts all the axons but does not cause excessive extra-axonal tissue damage, is optimal for evaluating regenerative potential of novel treatments.

Another important consideration in evaluating regenerative potential of treatments is the delivery system. For efficient targeting right after injury, we and others typically inject AAV2 intravitreally 2 weeks before ONC to express mRNA or shRNA^13,22,24,31,32,45,46^. Although as with any gene therapy the level of expression from AAV2 is difficult to control, interpretation of the effect is straightforward because regenerating axons are clearly quantifiable. Therefore, this is an appropriate delivery system for investigating regenerative potential of a treatment and is widely used in the field (see references above). Moreover, intravitreally injected AAV2 has been successfully tested in the human eye for treating Leber congenital amaurosis^47,48^ and Leber’s hereditary optic neuropathy^49,50^. Therefore, prospective findings using AAV2 have a clear path to clinic for treating optic neuropathies. Although pre-treatment in this model is not similar to a clinical setting, where treatment is delivered post-injury, the delay in efficient targeting that is intrinsic to current AAV2 systems necessitates pre-treatment in order to evaluate whether a treatment has the potential to stimulate axon regeneration. Injecting AAV2 after ONC, similar to a clinical setting, would hinder the ability to assess regenerative potential of a specific molecular target, as this delivery method is slow. The delayed onset of targeting that is intrinsic to current AAV2 systems is a matter of delivery system. Bioengineers are currently actively advancing drug delivery methods (including improved viruses and nanotechnologies) to overcome this limitation, which could facilitate faster and better controlled targeting post-injury. However, for the study of regenerative potential of molecular targets, pre-treatment is more appropriate, until reliable faster delivery systems become available to researchers. With the current AAV2 systems, post-injury tests are justified only after full-length axon regeneration and ensuing recovery of function has been achieved with pre-treatment. Administering AAV2 post-injury, prior to establishing whether or not it has sufficient regenerative potential without the confounding delay, may result in false-negative type II error, due to delivery system limitation rather than because the molecular target itself is not promising.

While pre-treatment is needed in this model system for evaluating regenerative potential of a specific target, it may also potentially promote axonal resistance to injury and lead to axoplasmic recovery (rather than regeneration *per se*). For example, axoplasmic recovery occurred in a glaucoma model when increased IOP was normalized^30^. Although the axons may have never been physically disrupted in that study, it still raises a theoretical possibility that pre-treatment may promote axonal resistance to ONC and lead to axoplasmic recovery. In such a hypothetical scenario, CTB anterograde axonal tracer would label the full-length of an axon because the axon would not be regenerating, as it had never been physically disrupted by the injury in the first place. Therefore, since prior studies have shown that even with pre-treatment it takes over 2 weeks for the axons to regenerate past the optic chiasm^22–24,33,51^, the absence of intact full-length axons at distal from the injury sites along the optic nerve (at 2 weeks after ONC) demonstrates that all the axons were disrupted, and that those axons that are detected along various distances of the optic nerve beyond the injury site (but that are not full-length) have in fact regenerated and not just recovered. If, unexpectedly, full-length axons are detected at 2 weeks after ONC, then even an earlier time-point should be tested. Moreover, testing at 2 time-points after injury and detecting that the distance at which the axons are detected has increased over time would not only further reaffirm that the axons are regenerating, but also show that axons have not stalled growth by 2 weeks. As noted above, many of the axons that have been stimulated to regenerate by different treatments arrest growth at various distances along the optic nerve and optic tract^12,22–24,33,45,51,52^, therefore, testing for progressive growth over time is important not only to control for axonal sparing or axoplasmic recovery, but also in order to evaluate regenerative potential of the treatment and to gain insights into the underlying mechanism. For example, we found that treatment with myr-Set-β increased both the distance axons regenerated and the number of regenerating axons from 2 to 3 weeks^45^, whereas co-treatment with anti-Klf9 shRNA and Zinc chelating TPEN increased the distance axons regenerated, but not their number, from 2 to 6 weeks^24^. While progressive axonal growth is therapeutically promising, treatments that lead to short-term regeneration that stops by 2 weeks may be important for the initiation but not necessarily the elongation stage of axonal growth (which in adult mammals may also be associated with the threshold for stalling growth in response to signaling by extracellular inhibitory molecules). Therefore, both types of mechanisms (initiation and elongation of axon growth) need to be investigated, and treatments that stimulate them independently need to be tested for possible synergistic effect in promoting more robust regeneration than each alone.

In sum, our study demonstrated that success of regenerative treatments depends on the preservation of extra-axonal tissue environment, and that upregulation of the axon growth-inhibitory molecules rather than tissue atrophy may underlie reduced regeneration after more severe extra-axonal tissue damage. We also showed how inefficient ONC could lead to axonal sparing and ways to control for this issue, as well as how to control for potential axoplasmic recovery without needing excessively harsh ONC. Finally, we argue for not confounding evaluation of regenerative potential of novel experimental treatments by administering AAV2 post-injury using current, slow delivery/targeting systems.

## Methods

### Animal Use, Surgeries, and Intraocular Injections

All animal studies were performed at the University of Connecticut Health Center with approval of the Institutional Animal Care and Use Committee and of the Institutional Biosafety Committee, and performed in accordance with the ARVO Statement for the Use of Animals in Ophthalmic and Visual Research. Mice were housed in the animal facility with a 12-h light/12-h dark cycle (lights on from 7:00 AM to 7:00 PM) and a maximum of five adult mice per cage. The study used wild-type 129S1/SvImJ mice (The Jackson Laboratory). Optic nerve surgeries and intravitreal injections were carried out on male mice 10–12 weeks of age (average body weight, 22–27 g) under general anesthesia, as described previously^22,24,53^.

Virus (3 μl per eye) was injected intravitreally, avoiding injury to the lens, in 10-week-old mice, 2 weeks prior to optic nerve surgery. This lead time allowed for sufficient transduction and expression of the shRNA to knockdown expression of Pten in RGCs at the time of ONC. The AAV2 virus expressing anti-Pten shRNAs (target sequences are as follows: 5′-GCAGAAACAAAAGGAGATATCA-3′, 5′-GATGATGTTTGAAACTATTCCA-3′, 5′-GTAGAGTTCTTCCACAAACAGA-3′, and 5′-GATGAAGATCAGCATTCACAAA-3′) titer was 1 × 10^12^ GC/mL (Cyagen Biosciences, Inc.).

Investigators performing the surgeries and quantifications were masked to the group identity by another researcher until the end of the experiment, and the animals that received AAV2 anti-Pten shRNA injections were randomly selected for 1 or 5 seconds ONC. A few animals that developed cataract in the injured eye were excluded.

### Histological Procedures

Standard histological procedures were used, as described previously^22,24,31,53^. Briefly, anesthetized mice were transcardially perfused with isotonic saline followed by 4% paraformaldehyde (PFA) at 2 weeks after optic nerve injury, optic nerves were dissected, postfixed 2 hours, and transferred to 30% sucrose overnight at 4 °C. The optic nerves were embedded in OCT Tissue Tek Medium (Sakura Finetek), frozen, cryostat-sectioned longitudinally at 14 µm, immunostained on coated glass slides, and then mounted for imaging. For immunostaining, tissue sections were blocked with the appropriate sera, incubated overnight at 4 °C with primary antibodies described in the main text, then washed three times, incubated with appropriate fluorescent secondary antibodies (1:500; Alexa Fluor, Life Technologies) overnight at 4 °C, washed three times again, and mounted. Images were acquired using fluorescent microscope (Zeiss, AxioObserver.Z1) and processed using ZEN software (Zeiss) with a deconvolution module for *z*-sacks (representative *z*-planes were extracted after deconvolution for representative images).

### Quantitation of Axons, CSPG levels, and Retinal Thickness

Axons were visualized at 2 weeks after optic nerve injury by immunostaining with the anti-CTB antibody (1:500, rabbit polyclonal; GenWay, #GWB-7B96E4) for the cholera toxin B (CTB, 1% in 3 μl PBS; List Biological, #103B) fragment, which was injected intravitreally 1 day prior to sacrifice. Sections were examined for possible axon sparing; no spared axons were found in controls and no evidence of axon sparing was found in experimental conditions (i.e., at 2 weeks after injury no axons were found at distal from the injury region of the optic nerve). Regenerated axons (defined as fibers continuous for >100 µm, which are absent in controls and are discernible from background puncta and artefactual structures), were counted manually using fluorescent microscope (Zeiss, AxioObserver.Z1) in at least 4 longitudinal sections per optic nerve at 1 mm, 1.5 mm, 2 mm, and 3 mm distances from the injury site (identified by the abrupt disruption of the densely packed axons near the optic nerve head, as marked by an asterisk in Figs 1 and 3), and these values were used to estimate the total number of regenerating axons per nerve, as described^22,24^. CSPG levels in uninjured optic nerves or along the injury site at 2 weeks after optic nerve injury were visualized by immunostaining in parallel with the anti-CSPG antibody (1:600, mouse IgG1; Millipore, #MAB5284). DAPI was used (1:2000) to label nuclei. Images were acquired using fluorescent microscope (Zeiss, AxioObserver.Z1) as above, and average fluorescent signal intensity was measured at different distances along the injury site, or in uninjured optic nerve in the same regions, using ImageJ software plugin Plot Profile. Retinal tissue thickness was quantified by measuring the distance between the ganglion cell layer (GCL) and the outer nuclear layer (ONL), visualized by DAPI staining (1:2000) in the retinal flatmounts from untreated mice 2 weeks 1 s or 5 s ONC. Each retina was sampled in the ventral, dorsal, lateral, and nasal regions around the optic nerve head. The measurements of retinal thickness were then averaged per eye. Retinal flatmounts were imaged using confocal microscope (Zeiss, LSM 880); *Z*-stocks with 0.5 µm intervals were rotated in 3D viewer (ZEN software, Zeiss) to create digital cross-sections of the retina, shown in the representative images.

### Statistical analyses

All tissue processing, quantification, and data analysis were done masked throughout the study. Sample sizes were based on accepted standards in the literature and our prior experiences. Sample size (*n*) represents total number of biological replicates in each condition. All experiments included appropriate controls. No cases were excluded in our data analysis, although a few animals that developed cataract in the injured eye were excluded from the study and their tissues were not processed. The data was analyzed by ANOVA with Repeated Measures and a posthoc LSD test, or Independent Samples *t*-Test, as indicated (SPSS). Data are presented as Means ± SEM. All differences were considered significant at *p* < 0.05.
